# The timing of umbilical cord clamping at birth: physiological considerations

**DOI:** 10.1186/s40748-016-0032-y

**Published:** 2016-06-13

**Authors:** Stuart B. Hooper, Corinna Binder-Heschl, Graeme R. Polglase, Andrew W. Gill, Martin Kluckow, Euan M. Wallace, Douglas Blank, Arjan B. te Pas

**Affiliations:** The Ritchie Centre, Hudson Institute for Medical Research, Melbourne, Australia; Department of Obstetrics and Gynaecology, Monash University, Melbourne, Australia; Centre for Neonatal Research and Education, The University of Western Australia, Crawley, WA 6008 Australia; Department of Neonatology, Royal North Shore Hospital and University of Sydney, Sydney, NSW 2065 Australia; Neonatal Services, The Royal Women’s Hospital, Melbourne, Australia; Department of Neonatology, Leiden University Medical Centre, Leiden, The Netherlands

**Keywords:** Delayed umbilical cord clamping, Birth, Neonatal cardiovascular transition, Umbilical artery flow, Umbilical venous flow

## Abstract

While it is now recognized that umbilical cord clamping (UCC) at birth is not necessarily an innocuous act, there is still much confusion concerning the potential benefits and harms of this common procedure. It is most commonly assumed that delaying UCC will automatically result in a time-dependent net placental-to-infant blood transfusion, irrespective of the infant’s physiological state. Whether or not this occurs, will likely depend on the infant’s physiological state and not on the amount of time that has elapsed between birth and umbilical cord clamping (UCC). However, we believe that this is an overly simplistic view of what can occur during delayed UCC and ignores the benefits associated with maintaining the infant’s venous return and cardiac output during transition. Recent experimental evidence and observations in humans have provided compelling evidence to demonstrate that time is not a major factor influencing placental-to-infant blood transfusion after birth. Indeed, there are many factors that influence blood flow in the umbilical vessels after birth, which depending on the dominating factors could potentially result in infant-to-placental blood transfusion. The most dominant factors that influence umbilical artery and venous blood flows after birth are lung aeration, spontaneous inspirations, crying and uterine contractions. It is still not entirely clear whether gravity differentially alters umbilical artery and venous flows, although the available data suggests that its influence, if present, is minimal. While there is much support for delaying UCC at birth, much of the debate has focused on a time-based approach, which we believe is misguided. While a time-based approach is much easier and convenient for the caregiver, ignoring the infant’s physiology during delayed UCC can potentially be counter-productive for the infant.

## Background

The transition from fetal to newborn life represents one of the greatest physiological challenges that any human will encounter. Once the umbilical cord is clamped, infants must clear their airways of liquid to allow the onset of pulmonary gas exchange and the cardiovascular system must undergo a major structural and functional re-organisation [1]. Although it is well recognized that the cardiovascular transition at birth is triggered by lung aeration [2, 3], the question of how umbilical cord clamping (UCC) influences this relationship is unclear [1]. It is widely assumed that UCC at birth is an innocuous act, but many have argued that this assumption is false and, if done too early, can deprive the infant of vital blood volume during early newborn life [4, 5]. The aim of this review is to discuss the physiology of umbilical cord clamping and the circumstances that would facilitate placental transfusion if UCC is delayed.

**Fig. 1:**
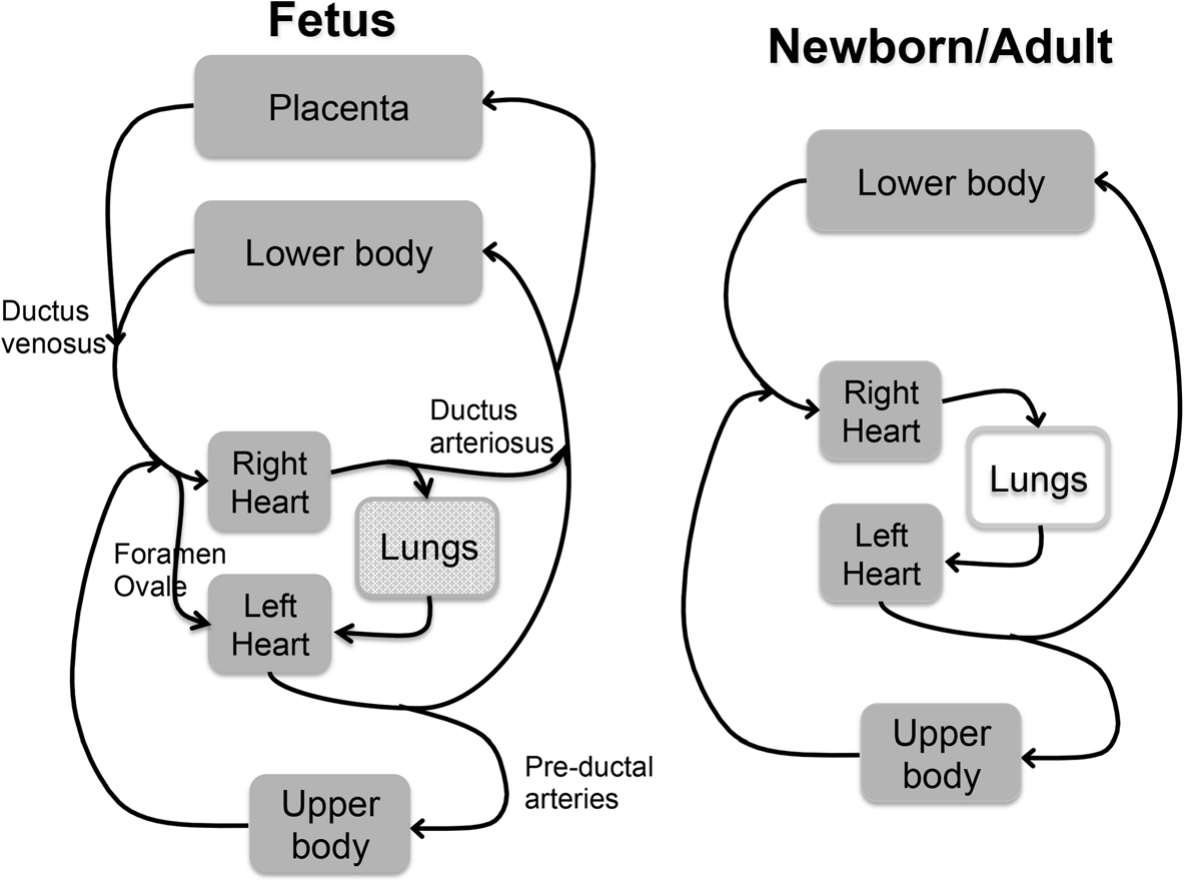
A schematic of both the fetal and newborn/adult circulations, showing the anatomical relationships, and connections (in the fetus), between the pulmonary and systemic circulations. Before birth, the majority of venous return to the left heart is derived from the placenta, which passes through the ductus venosus and foramen ovale, because pulmonary blood flow is low. After birth, following clamping of the umbilical cord, the supply of preload for the left heart switches from the placenta to the pulmonary circulation

At birth, lung aeration triggers a functional re-organisation of the infant’s circulation, largely by stimulating an increase in pulmonary blood flow (PBF) [1]. From a teleological perspective, linking these physiological events is logical. As lung aeration can only occur after birth and is a pre-requisite for newborn survival, it is an ideal trigger for initiating the physiological changes that underpin the transition to newborn life. In this context, when trying to understand and devise strategies that assist the infant in making the best possible transition to newborn life, it is important to understand the central role that lung aeration plays in this process. Indeed, neonatologists have long recognized that, at birth, ventilation is the key to newborn resuscitation. It not only increases oxygenation, but also increases the infant’s heart rate and cardiac function by stimulating an increase in PBF [3]. An increase in PBF restores the preload required to maintain cardiac output after birth (Fig. 1), which is lost upon umbilical cord clamping (UCC) [6, 7]. In view of the central role that lung aeration plays in the cardiovascular transition at birth, it is also likely to have a major impact on placental to infant blood transfusion when UCC is delayed. However, until recently all arguments about delayed UCC have simply focused on the time that UCC should be delayed with little or no reference to the infant’s transitional physiology [5]. In this review, we will argue that there is little or no justification for delaying UCC for a set period of time after birth and will discuss physiological factors that may provide a more rational determinant for when UCC should occur after birth.

### A historical perspective

It has long been recognized that UCC is not just a symbolic separation of the infant from the mother, but can have a major impact on the infant’s well-being after birth. Indeed, the argument of when the umbilical cord should be clamped extends back centuries, at least to Aristotle in 300 BC. In 1801, Erasmus Darwin suggested that, *‘Another thing very injurious to the child, is the tying and cutting of the navel string too soon; which should always be left till the child has not only repeatedly breathed but till all pulsation in the cord ceases. As otherwise the child is much weaker than it ought to be*’ [8]. In this commentary, Darwin highlights the link between breathing and the timing of UCC, indicating that he considered the two events to be closely linked and to impact on the infant’s well being. However, the debate about the timing of UCC at birth has largely overlooked the impact that pulmonary ventilation may have in this process.

Until recently, the benefits of delayed UCC at birth were thought to only involve placental-to-infant blood transfusion. This debate was sparked by a series of studies demonstrating a time dependent “transfusion” of blood into the infant if UCC is delayed for up to 3 mins after birth [9–11]. However, in view of recent studies (see below), it is now time to question whether this concept is an overly simplistic view of the factors controlling blood flow between the placenta and infant immediately after birth. These early studies used ^125^I-labeled albumen to measure blood volumes in infants that had their cords clamped at different times after birth [9, 11]. However, as we now know that blood flow in the umbilical arteries and veins during delayed UCC is regulated by a complex interplay of physiological factors, mostly respiratory [12], it is hard to envisage how a time-dependent increase in blood volume could be so consistently achieved. Measurements of increasing infant weight have provided additional evidence supporting the concept of placental-to-infant blood transfusion [13], but there is still considerable uncertainty as to the factors that influence this transfer and whether time is incidental or a significant factor [1].

Recent studies have now begun to investigate the many factors that may influence blood flow in the umbilical arteries and veins during delayed UCC and while there is the potential for placental-to-infant blood transfer, there is also a risk of infant-to-placenta blood transfer [12]. Thus, with regard to making recommendations about the timing of UCC, we need to better understand the factors regulating blood flow in the umbilical vessels after birth and identify the factors that influence the distribution of blood between the placenta and infant at this time. This must also include a better understanding of the physiology underpinning the cardiovascular transition at birth.

### The cardiovascular transition at birth: the effect of UCC before lung aeration

Before birth, blood flow through the lungs is low as the majority of blood exiting the right ventricle by-passes the lungs and enters the thoracic aorta via the ductus arteriosus (DA) [6, 7, 14]. As a result, pulmonary venous return is also low and only provides a small proportion of the preload required to maintain left ventricular output in the fetus (Fig. 1). Instead, much of the preload for the left ventricle during fetal life is derived from umbilical venous return [6, 7, 14]. This blood flows from the umbilical vein, via the ductus venosus, inferior vena cava and through the foramen ovale to directly enter the left atrium [14]. The preferential streaming of well-oxygenated umbilical venous blood through the ductus venosus and foramen ovale into the left atrium, gives rise to higher oxygenation levels in fetal preductal (vs postductal) arteries [14].

**Fig. 2:**
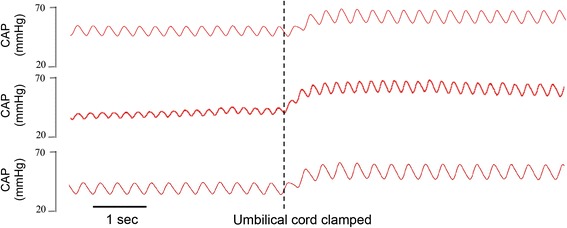
Effect of umbilical cord clamping (dotted line) on carotid arterial blood pressure (CAP) in three lambs. CAP increases by ~30 % in 4–5 heart beats [6]

**Fig. 3:**
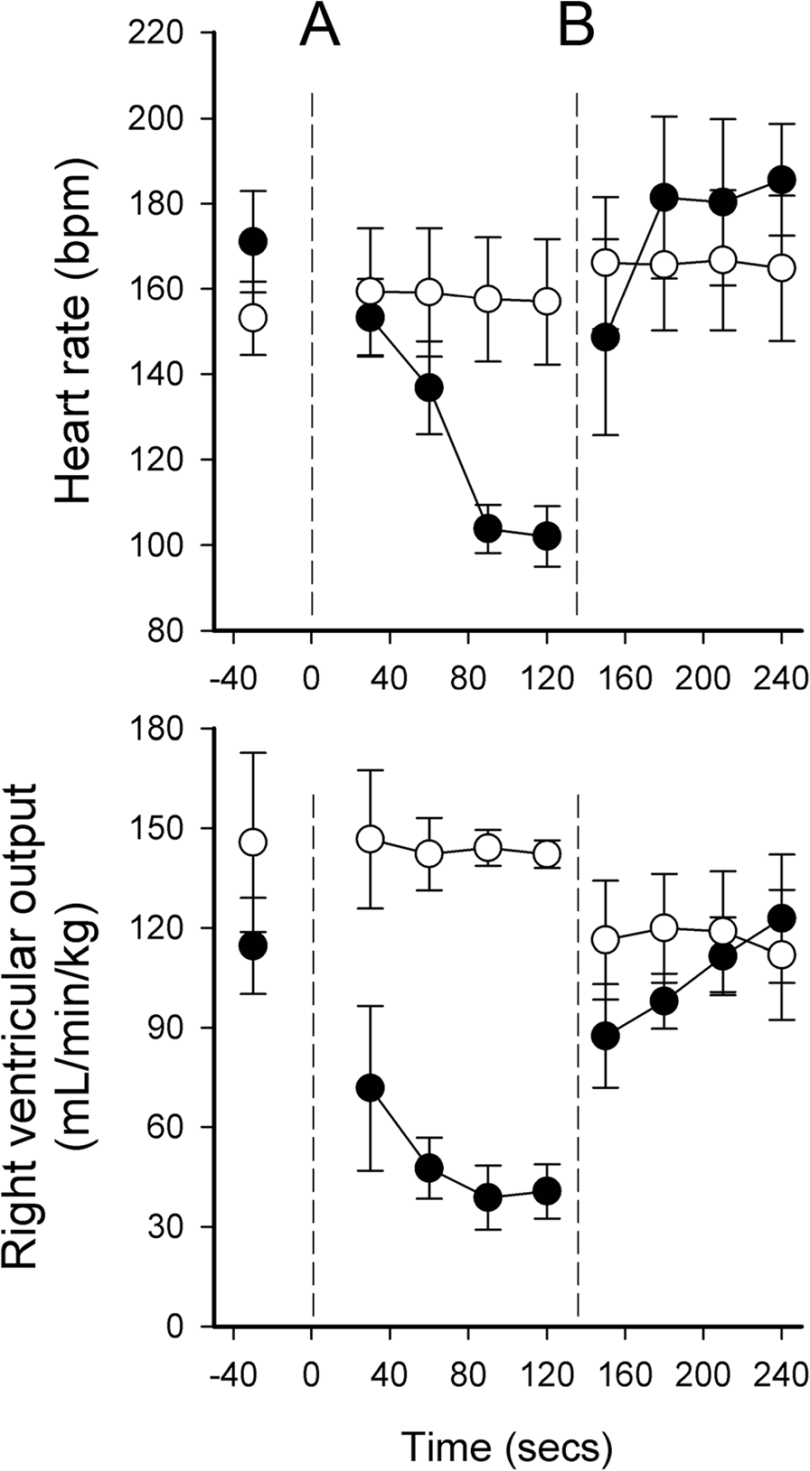
Heart rate and right ventricular output measured in newborn lambs that either had their umbilical cords clamped 1–2 mins before ventilation was commenced (clamp first; closed circles) or were ventilated and pulmonary blood flow allowed to increase before their cords were clamped (vent first; open circles). The broken line (**a**) indicates either when cord clamping occurred in the clamp first group or ventilation commenced in the vent first group. The broken line (**b**), indicates when either clamping occurred in the vent first group or when ventilation commenced in the clamp first group. Data were obtained from [6] and redrawn

While the fetal circulatory arrangement allows a relatively direct flow of oxygenated blood from the placenta into the left atrium, which is analogous to flow between the lungs and left atrium in adults (Fig. 1), UCC at birth can severely disrupt venous return and cardiac output [6, 7]. Indeed, UCC causes venous return to decrease by 30–50 %, which reduces preload and cardiac output by a similar amount [6, 7]. At the same time, total peripheral resistance increases with the loss of the low resistance placental circulation, which causes a rapid increase (30 % within 4 heart beats; Fig. 2) in arterial blood pressure [6, 15, 16]. No doubt this increase in afterload contributes to the decrease in cardiac output, which is reflected by both a decrease in stroke volume and a decrease in heart rate [6, 7] (Fig. 3). With regard to the latter, it is important to recognize that the low heart rates commonly observed at birth [17], even in normal term infants, may result from a loss of preload caused by UCC rather than from an acute hypoxic episode. Indeed, a recent study in rabbits has shown that ventilation with 100 % nitrogen also increases PBF and heart rate after birth [18]. Thus, an increase in oxygen is unlikely to be the only stimulus for the increase in heart rate at birth, which also likely involves an increase in PBF via an increase in preload [1].

**Fig. 4:**
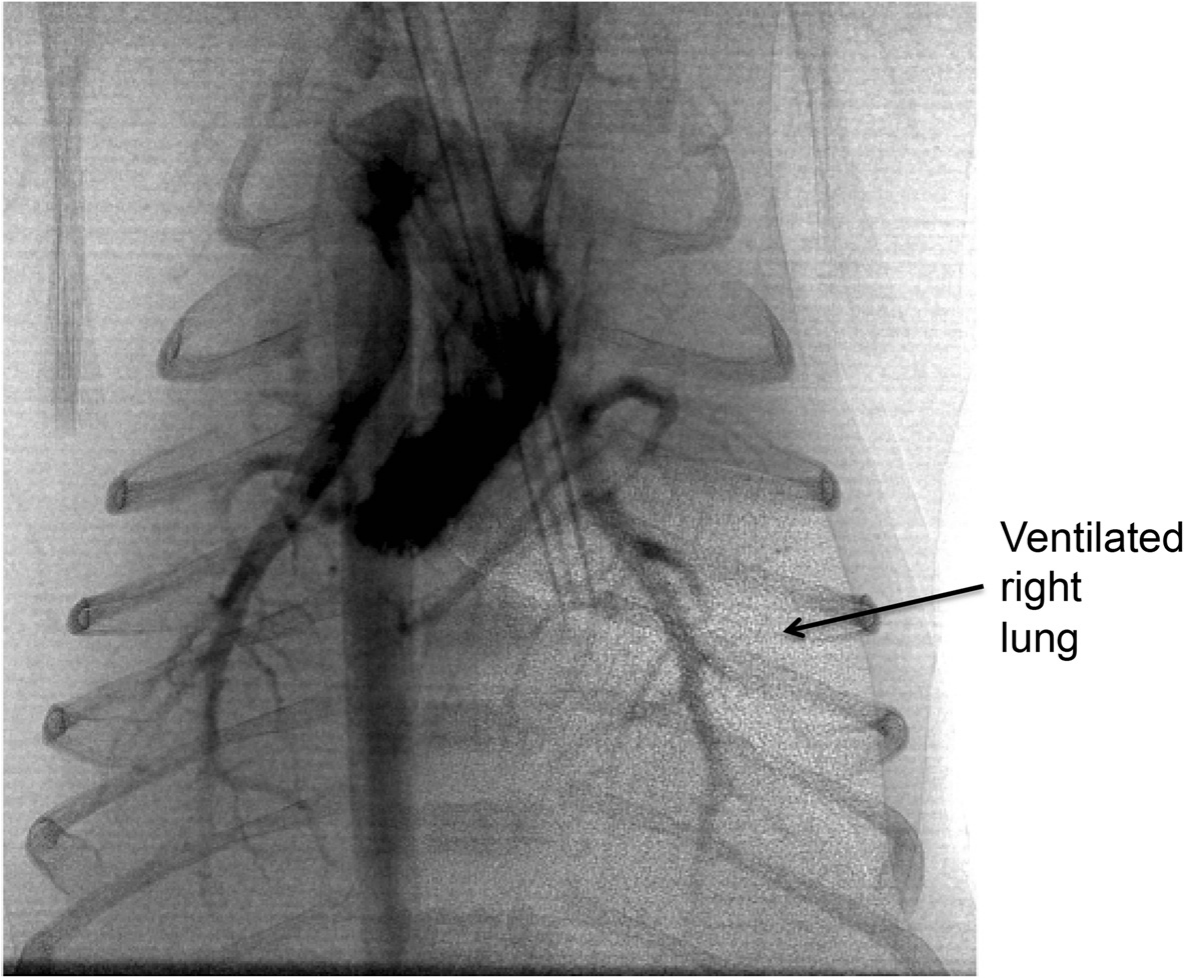
A combined angiographic and phase contrast X-ray image of a near term (30 days) rabbit kitten that was delivered by caesarean section and received unilateral ventilation of the right lung. Blood flow, as shown by the contrast agent in the pulmonary vessels, increases similarly in both the aerated right lung and the unaerated left lung

While the precise mechanisms by which lung aeration stimulates the increase in PBF at birth are still unclear [19], a recent imaging study has shown that lung aeration and the increase in PBF are not spatially related [20]. This study showed that partial lung aeration caused a global increase in PBF, leading to a large ventilation/perfusion mismatch in unaerated regions of the lung (Fig. 4). A follow up study showed that this global increase in PBF in response to partial lung aeration also occurs following ventilation with 100 % nitrogen [18]. These unexpected findings suggest that the dominant mechanisms involved are different to the mechanisms regulating regional PBF in the adult and that while oxygen must play a role [21], other mechanisms are also involved. Nevertheless, as PBF becomes the sole source of preload for the left ventricle after birth, PBF must increase shortly after UCC to replace umbilical venous return as the primary source of preload for the left ventricle [6]. As such, if there is a delay between UCC and the onset of lung aeration, the infant will not only be exposed to hypoxia, due to a lack of gas exchange, but also to a prolonged period of reduced or restricted cardiac output. As the primary physiological defense mechanism that is invoked during hypoxia is an increase and redistribution of cardiac output [22–24], this period of reduced cardiac output puts the infant at high risk of hypoxic/ischemic injury. On the other hand, if ventilation onset coincides with or immediately follows UCC, any reduction in cardiac output is likely to be brief and greatly reduced.

It is also important to consider how the infant’s physiology responds to UCC and the sudden reduction in cardiac output combined with a rapid (over 4 heart beats; Fig. 2) increase in arterial blood pressure (afterload) [6]. As the cerebral circulation is pressure passive over this time frame, this increase in pressure leads to an increase in cerebral blood flow in lambs. However, as cardiac output is also decreased, after ~60 s arterial blood pressure and cerebral blood flow also decrease before stabilizing (after ~2mins), presumably due to a baroreceptor mediated peripheral vasoconstriction [6]. Then after ventilation onset, the sudden increase in PBF restores left ventricular preload and increases cardiac output, leading to a second rapid increase in arterial blood pressure and cerebral blood flow (Fig. 3); increases in cardiac output have also been observed in human infants at birth [25, 26]. The net result of UCC followed by lung aeration after a brief delay (1–2 mins), are large fluctuations in arterial pressure and cerebral blood flow [6]. It is also interesting that right ventricular output rapidly increases with cardiac output following lung aeration, suggesting that left-to-right shunting through the foramen ovale may contribute to right ventricular preload at this time (Fig. 3).

Another consideration with regard to the impact of UCC at birth and the associated reduction in cardiac output, is the question of how PBF increases so rapidly and to such a large extent when right and left ventricular output are initially both low. The answer is partly due to the redirection of right ventricular output through the lungs, rather than through the DA, as pulmonary vascular resistance (PVR) decreases [6, 7, 14]. In addition, because UCC greatly increases peripheral vascular resistance, the decrease in PVR makes the lungs a lower resistance pathway for blood flow compared to the systemic circulation. As a result, blood flow through the DA reverses, leading to left-to-right shunting of blood from the aorta into the pulmonary artery, which makes a significant contribution to the increase in PBF [7]. While the net flow is left-to-right, instantaneous flow is bi-directional at different times throughout the cardiac cycle [7, 27]. This is because the pressure waves emanating from the left and right ventricles reach the pulmonary and aortic ends of the DA at different times after the onset of systole. During early systole, right ventricular contraction produces a pressure gradient across the DA that leads initially to right-to-left flow across the DA. However, as the pressure wave emanating from the left ventricle reaches the DA-aortic junction, the pressure gradient reverses, which produces left-to-right flow through the DA that is sustained throughout most of diastole. Interestingly, the net left-to-right flow through the DA leads to a partial left ventricle-lung-left ventricle short circuit [7]. Presumably this allows the two ventricles time (while the DA closes) to gradually balance their outputs after birth, which ensures that the left ventricle is not deprived of preload during this transitional period.

### Cardiovascular transition at birth: the effect of UCC after lung aeration

As the source of preload for the left ventricle must switch from umbilical venous return to pulmonary venous return after birth (Fig. 1), to avoid the loss of preload and reduction in cardiac output caused by UCC, it is logical to aerate the lungs and allow PBF to increase before UCC [1, 6, 28]. In this situation, the preload source can immediately switch from the umbilical circulation to the pulmonary circulation with little or no reduction in supply. As a result, there is no reduction in cardiac output and interestingly the normal increase in arterial blood pressure caused by UCC is greatly mitigated [6]. This is because there is an almost instantaneous reversal of blood flow though the DA, which changes from entirely right-to-left to mostly left-to-right following UCC [6, 7]. This indicates that the low resistance pulmonary circulation instantly becomes an alternative route for systemic blood flows and thereby greatly reduces the instantaneous increase in afterload caused by UCC.

Following lung aeration and before UCC, it would appear that there is a competitive interplay between blood flows entering the pulmonary and placental circulations. This is because, while the DA is patent, flow exiting the right and left ventricles can either enter the pulmonary circulation or continue down the aorta and enter the placental circulation (Fig. 1). Clearly the determining factor as to which circulation dominates will depend on the resistance in each circulation. At least initially, flow through the placental circulation appears to dominate as, while right-to-left DA flow is reduced following ventilation onset, no left-to-right flow occurs until after UCC [6]. However, as UCC occurred after only 2–3mins of positive pressure ventilation in that study and it takes up to 10 mins for PBF to peak (and PVR to reach a minimum) following ventilation onset [7, 29], it is possible that the pulmonary circulation dominates after longer periods of ventilation; we have recently observed this in lambs (unpublished observations). This effect may occur sooner and to a greater extent under conditions where the decrease in PVR is enhanced, for example if the infant makes deep inspiratory efforts [27], or when placental vascular resistance is increased, perhaps due to uterine contractions (see below). The logical extension to this concept is the suggestion that the re-direction of both right and left (via the DA) ventricular output into the pulmonary circulation, contributes to the reduction in umbilical artery blood flow after birth and to the eventual closure of these vessels (Fig. 1). In any event, these considerations highlight the fact that flow in the umbilical vessels is likely to be influenced by many interacting factors and whether this results in net placental to infant blood transfusion is not a forgone conclusion.

### Factors regulating umbilical blood flows during delayed cord clamping after birth

The concept that net placental to infant blood transfusion occurs if UCC is delayed for a set period of time assumes that umbilical venous flow will exceed umbilical arterial flow during this time. However, until recently, very little was known about the factors determining flow in the umbilical circulation following birth and before UCC. Indeed, it is unclear if flow in the umbilical arteries and vein are influenced by the same factors and to the same extent or are independently regulated, which is highly unlikely. Clearly, if delayed UCC is going to be recommended for clinical practice, it is important to identify the factors that regulate flow in both vessels after birth. This is needed to ensure that factors that promote infant-to-placental blood transfusion are avoided, as clearly this would be counterproductive and detrimental to the infant.

#### Gravity

While little consideration has been given to the factors determining flows in the umbilical arteries and vein during delayed UCC, there has been much discussion of factors that may influence net placental to infant blood transfusion [4, 30]. In particular, it has been proposed that gravity will assist blood transfusion if the infant is placed below the level of the introitus [10]. However, this assumes that the flows in the umbilical arteries and vein are independent and that gravity will increase flow in the vein but decrease flow in the arteries. We have recently examined this in lambs and found that placing the lamb below the ewe during delayed UCC reduces umbilical artery flow and initiates left-to-right shunting through the DA leading to an increase in PBF (unpublished observations). Logically, the higher pressure-head required for the heart to pump blood through the placenta, due to the vertical height difference between the heart and placenta, makes the lungs a lower resistance option than the placenta. This supports the concept that more blood remains in the infant with each heartbeat. However, placing the lamb below the placenta also reduced umbilical venous flow by a similar amount, indicating that flow into the placenta is a major determinant of flow out of the placenta. As such, unless the resistance of the placental or infant’s circulation markedly changes at this time, it is hard to understand how placing the infant below the mother will influence net placental to infant blood transfusion. This is consistent with the findings of a recent clinical trial, which found that placing an infant at the same height or above the introitus does not effect placental to infant blood transfusion, with both groups apparently receiving a similar transfusion of 50–55 mL [31].

#### Uterine contractions

Another factor proposed to increase placental to infant blood transfusion is uterine contractions. It has been suggested that uterine contractions physically “squeeze” blood out of the placenta and into the infant [9, 30], but this suggestion is not consistent with the changes in umbilical blood flows that occur during labour [32]. While uterine contractions have a major impact on umbilical blood flows, they are primarily thought to cause a pressure-induced, differential reduction in flow in both vessels as well as a reduction in uterine flow [32]. During the contraction, the lower pressure umbilical veins are thought to close earlier than the higher pressure umbilical arteries at the onset of contraction and the venous vessels also open later than the arteries towards the end of the contraction [32]. As such, there is the potential for placental blood accumulation during a contraction. The release of this blood back into the fetal circulation at the end of the contraction is thought to give rise to the small tachycardia that follows a uterine contraction [32].

We have recently examined the effect of oxytocin-induced contractions on umbilical artery and venous blood flows in lambs during delayed UCC (unpublished observations). We found that during the contraction, umbilical venous flow ceased and that while pulsatile flow continued in the umbilical artery, the flow was greatly reduced resulting in retrograde flow during diastole. A similar flow pattern in the umbilical artery has been observed in infants during delayed UCC, although it was unknown whether this flow pattern coincided with a uterine contraction [12]. Uterine contractions were also found to increase arterial blood pressure in lambs, which is consistent with it causing an increase in placental vascular resistance as observed with UCC [6]. Thus, rather than facilitating placental to infant blood transfusion, uterine contractions appear to more closely replicate partial UCC and thereby restrict venous return, reduce cardiac output and increase afterloads. Thus, perhaps the administration of oxytocin-like compounds to mothers immediately after birth, to reduce the risk of post-partum haemorrhage, should be delayed until after UCC.

#### Spontaneous breathing

**Fig. 5:**
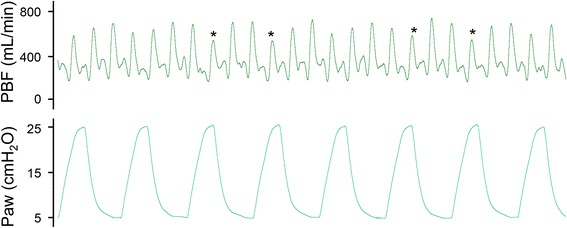
Simultaneous pulmonary blood flow and airway pressure recordings made in a ventilated newborn lamb. Note that whenever the peak airway pressure coincides with systole, the systolic peak in pulmonary blood flow is reduced, particularly compared with the systolic peaks that coincide with periods when airway pressure is low between inflations. The reductions in pulmonary blood flow result from transient increases in pulmonary vascular resistance caused by the increase in airway pressure. Asterisks indicate good examples of peak pulmonary blood flow reductions coinciding with peak inflation pressures

The recently published experimental studies on delayed UCC only examined the physiological effects of positive pressure ventilation (PPV) [6], whereas the effects of spontaneous breathing are likely to be different. It is well established that increasing intra-thoracic pressure reduces PBF by increasing PVR and if the pressures are sustained [33, 34], venous return and preload are also reduced, resulting in a reduction in cardiac output. The inhibitory effect of positive airway pressures on PBF also occurs on an inflation-by-inflation basis during PPV. In lambs, when the systolic peak in PBF is in phase with the peak inflation pressure, the amplitude is reduced, whereas when they are out of phase, peak PBF is increased (Fig. 5). The pressure related decrease in PBF is thought to result from alveolar pressurization and expansion causing compression of pulmonary capillaries that lie between adjacent alveoli, in the alveolar walls [34–36]. On the other hand, spontaneous breathing in infants results in a reduction in intra-thoracic pressure, which facilitates venous return and transiently increases both PBF and left-to-right DA shunting [37]. In contrast to positive pressure inflations, when inspiration coincides with systole, peak PBF is increased, which is thought to result from peri-alveolar capillary expansion and a reduction in peri-alveolar tissue pressures [38]. This is caused by lung expansion resulting from chest wall expansion, whereby the expanding forces are projected from the visceral pleura into the alveoli, rather than commencing within the alveoli and expanding out.

Recent studies in infants have shown that spontaneous breathing at birth, particularly deep inspiratory efforts, have a major impact on PBF and blood flow through the DA, which is consistent with a large reduction in PVR during inspiratory efforts [27]. In normal spontaneously breathing infants, the proportion of right-to-left shunting of blood through the DA was found to reduce with time whereas the proportion of left-to-right shunting increased along with an increase in left ventricular output [25, 39]. Large inspiratory efforts associated with crying were found to cause a large increase in left-to-right DA shunting [27].

In view of the finding that spontaneous breathing has a large impact on PBF and DA shunting, it is not surprising that it also influences umbilical venous and arterial blood flows [12]. A recent study in infants has for the first time reported blood flows in the umbilical vessels during delayed UCC and identified some of the factors regulating blood flow between the infant and placenta after birth [12]. As predicted, the flow pattern was not uni-dimensional or uni-directional, with many factors determining blood flow in the cord. Flows continued for longer than previous thought and were found to cease in the umbilical vein before they ceased in the artery in approximately one third of infants; this could result in a net loss of blood from the infant [12]. Umbilical venous flow was very dependent upon the breathing cycle with flow entering the infant predominantly during inspiration and ceasing during expiration as well as during crying. The latter is not surprising as crying causes the abdomen and chest to pressurize and so umbilical venous pressure would have to substantially increase before blood could enter the infant. Similarly, crying was found to greatly reduce umbilical artery flow and in some cases, causes it to briefly cease [12]. In addition, as indicated above, periods of bidirectional flow was observed, which was thought to coincide with uterine contractions. Taken together, these findings clearly demonstrate that blood flow between the placenta and infant after birth is complex, is not unidirectional and is influenced by a number of factors. The dominant factors likely include uterine contractions and whether or not the infant is breathing or crying. Time from birth has little relevance to these factors except that with increasing time there is a higher chance that the infant will have commenced breathing.

### Summary

Recent experimental evidence and observations in human infants have provided compelling evidence to demonstrate that time is largely incidental and is not a major determining factor of net placental-to-infant blood transfusion after birth. It is also clear, that there are many factors that influence blood flow in the umbilical vessels after birth, which depending on the dominating factors could potentially result in infant-to-placental blood transfusion. Studies investigating factors that influence umbilical artery and venous blood flows before UCC have found that the most dominant factors are lung aeration, spontaneous inspirations, crying and uterine contractions. While it is still not entirely clear whether gravity differentially alters umbilical artery and venous flows, the available data suggests that its influence, if present, is minimal. The physiological consequences of umbilical cord “milking” have not been raised in this review, largely because there is very limited experimental data detailing the physiological effects on the newborn and its circulatory transition at birth. Despite this, numerous trials have been conducted expecting it to increase blood volumes and duplicate the advantages of delayed UCC without really understanding the impact that it will have on the infant’s physiology. As such physiological studies on umbilical cord milking are urgently needed.

## Conclusions

The debate about when the umbilical cord should be clamped after birth has simply focused on the potential for a time-dependent net placental to infant blood transfusion. However, as there are many factors that influence blood flow in the umbilical arteries and veins immediately after birth, some infants maybe at risk of losing blood volume. If UCC is to be delayed, there is now very good evidence demonstrating that the timing of UCC should be based on the infant’s physiology, rather than on a stopwatch. In particular, whether the infant is breathing or not. Aerating the lungs increases PBF, allowing pulmonary venous return to immediately replace umbilical venous return as the primary source of preload, which has the effect of stabilizing the circulation as it transitions after birth.

## Abbreviations

DA, Ductus arteriosus; PBF, Pulmonary blood flow; PPV, Positive pressure ventilation; PVR, Pulmonary vascular resistance; UCC, Umbilical cord clamping.
